# The association between elevated serum uric acid levels and islet β-cell function indexes in newly diagnosed type 2 diabetes mellitus: a cross-sectional study

**DOI:** 10.7717/peerj.4515

**Published:** 2018-03-09

**Authors:** Yimeng Hu, Jie Liu, Huiqiong Li, Hui Zhu, Linjie Liu, Yin Yuan, Jing Chen, Ye Wang, Xuemei Hu, Yancheng Xu

**Affiliations:** 1 Department of Endocrinology, Zhongnan Hospital of Wuhan University, Wu Han, Hu Bei, China; 2 Department of Endocrinology, Gezhouba Group Central Hospital, Yi Chang, Hu Bei, China; 3 Department of Gerontology, General Hospital of the Yangtze River Shipping, Wu Han, Hu Bei, China; 4 Department of Integrated Wards, Zhongnan Hospital of Wuhan University, Wu Han, Hu Bei, China

**Keywords:** Serum uric acid, Type 2 diabetes mellitus, Insulin secretion, Insulin resistance

## Abstract

**Background:**

Serum uric acid (UA) has been reported as a risk factor for type 2 diabetes mellitus (T2DM). However, whether serum UA is associated with insulin resistance and insulin secretion, and the effect of gender on it in the case of the existed association, both remain undefined.

**Methods:**

A cross-sectional study was designed and performed, which enrolled a total of 403 newly diagnosed T2DM patients (mean age, 50.21 ± 13.34 years (62.5% males)). Clinical characteristics and islet function indexes of all participants were analyzed based on gender-specific tertiles of serum UA levels. In addition, multiple linear regression analysis was conducted to investigate covariates associated with islet function indexes.

**Results:**

The mean levels of serum UA were 331.05 μmol/L (interquartile range (IQR): 60.6, 400.9 μmol/L) and 267.9 μmol/L (IQR: 204.7, 331.9 μmol/L) in men and women, respectively. The values of insulin secretion indexes involving AUCins30/glu30, AUCins120/glu120 and total insulin disposition index (DI120) in females were significantly higher than those in males. Apart from the homeostasis model assessment insulin resistance of men, serum UA was positively associated with insulin secretion and insulin resistance indexes both in men and women. Multivariable linear regression analysis showed serum UA exerted an independent impact on insulin secretion in females, but not on insulin resistance. In males, islet function was simultaneously affected by serum UA age, body mass index (BMI), and serum lipids.

**Conclusion:**

Serum UA harbored a positive correlation with insulin secretion and insulin resistance indexes in newly diagnosed T2DM patients, which was influenced by gender, BMI, serum lipids. Hence, serum UA may be considered as a predictor for islet function in clinical practice.

## Introduction

Type 2 diabetes mellitus (T2DM) is one of the most prevalent chronic diseases at present, affecting 415 million adults worldwide (Ogurtsova et al., 2017). The overall prevalence of diabetes is estimated to be 11.6% in the Chinese adult population (Xu et al., 2013). Previous studies have found that serum uric acid (UA) is a potential risk factor for many chronic diseases, such as cardiovascular diseases (Du et al., 2014), hypertension (Kuwabara et al., 2018), and kidney diseases (Zoppini et al., 2012). UA is the product of purine metabolism in humans whose serum concentration depends on dietary intake of purine-containing food and on excretion by urine. Without exception, elevated serum UA has been considered to be closely related to components of metabolic syndrome (MS) in nondiabetic and T2DM populations; thus, the assessment of UA could provide useful information for predicting MS (Nan et al., 2008; Bonakdaran & Kharaqani, 2014). The idea of serum UA as an independent risk factor for T2DM has been debated due to its association with other diabetic risk factors such as obesity (Nan et al., 2007). Yet, an increasing number of studies have provided evidence that UA is positively associated with serum glucose in healthy and diabetic individuals (Viazzi et al., 2011; Li et al., 2015; Xu et al., 2016).

As is known, progressive deterioration of islet β-cell function and insulin resistance are well-recognized symbols during the development of T2DM in adults. Recent findings suggest that serum UA is the precursor of T2DM rather than the consequence of insulin resistance (Juraschek et al., 2014). Although a number of animal and clinical studies have confirmed that UA plays an important role in the onset of diabetes via oxidative stress and inflammatory processes (Yiginer et al., 2008; Keenan et al., 2012), the association between serum UA with islet β-cell function is still poorly understood. A previous research has found elevated levels of UA increased β-cell apoptosis by activating the NF-κB–iNOS–NO signaling pathway both in vivo and in vitro (Jia et al., 2013). In a clinical study, serum UA has been reported to show a positive relationship with the total phase of insulin secretion (Robles-Cervantes et al., 2011), which is consistent with the conclusion of the Ling’s study (Ling et al., 2012). Together, these studies from different population samples have established an association between serum UA and basal insulin secretion. However, it remains unknown of the effect of serum UA level on different phases of insulin secretion ability and insulin sensitivity in newly diagnosed T2DM patients. And whether there are some other factors collectively influencing islet function requires further investigation.

Therefore, we conducted a cross-sectional study to (a) link serum UA levels to islet function indexes in newly diagnosed T2DM patients; (b) examine whether the serum UA levels in T2DM are influenced by age, gender, or body mass index (BMI); and (c) further explore the impact of high serum UA levels on blood pressure, blood lipid profiles, and renal function indicators.

## Methods

### Subjects

In this study, we included Chinese participants aged 20–85 years old who were newly diagnosed with untreated T2DM at Zhongnan Hospital of Wuhan University during 2010–2017. The diagnosis of T2DM was according to the criteria of the World Health Organization (WHO) (fasting plasma glucose (FBG) ≥7.0 mmol/L or 2 h-postprandial plasma glucose (2hPBG) >11.1 mmol/L) (Alberti & Zimmet, 1998). A 75 g oral glucose tolerance test was performed to assess glucose homeostasis and insulin release. To avoid confounding variables, we excluded patients with diabetic micro- and macro-vascular complications, thyroid disease, Cushing syndrome, liver cirrhosis, pheochromocytoma, renal failure, malignant tumors, and those who were pregnant or currently taking UA-lowering therapy. A total of 403 participants was enrolled in this study (mean age, 50.21 ± 13.34 years (62.5% males)). The study protocol was approved by the Ethics Committee of Wuhan University, and all participants signed written informed consent (Ethical Application Ref: 2016019).

### Measurements

Study participants were inquired about their age, family history, alcohol consumption, smoking, and past medical conditions. BMI was calculated as weight in kilograms divided by the height in meters squared (kg/m^2^). Normal weight, overweight, and obesity were defined as BMI < 23 kg/m^2^, BMI: 23.0–24.9 kg/m^2^, and BMI ≥ 25 kg/m^2^ for Asian population, respectively, according to Asian-Pacific WHO BMI criteria (WHO Expert Consultation, 2004).

Participants’ seated blood pressure was measured by trained nurses on the left arm with a sphygmomanometer upon initial examination. Overnight fasting blood samples were collected from the antecubital vein of each individual to test for serum total cholesterol (TC), triglycerides (TG), high-density lipoprotein-cholesterol (HDL-c), low-density lipoprotein-cholesterol (LDL-c), blood urea nitrogen (BUN), serum creatinine (Scr), and UA levels. All parameters were analyzed by the standardized enzymatic method.

### OGTT and insulin releasing test

After a 12–16 h, overnight fast, patients were administered with a 75 g glucose solution. Blood samples were collected from the antecubital vein at 0, 30, 60, 120, and 180 min intervals to measure plasma glucose (FBG, Glu30, Glu60, Glu120, Glu180) and serum insulin (Ins0, Ins30, Ins60, Ins120, Ins180). Plasma glucose was evaluated with the glucose oxidase method, and the serum insulin level was determined by chemiluminescent enzyme immunoassay.

### Calculation of islet function indexes

Insulin sensitivity was evaluated based on the homeostasis model assessment insulin resistance (HOMA-IR) and insulin sensitivity index (ISI) composite (Matsuda & DeFronzo, 1999). HOMA-β provided an index to measure basic insulin secretion, whereas the area under the curve of insulin-to-glucose ratio (AUC insulin/glucose) reflected insulin response to glucose at different times. Here, the value of the AUC was calculated using the trapezoid method. In consideration of the effect of insulin sensitivity on these indexes, several researchers developed the early-phase insulin disposition index (DI30) and the total insulin disposition index (DI120) to assess insulin secretion with respect to insulin sensitivity (Utzschneider et al., 2009; Ram et al., 2015).

To evaluate islet β-cell function, the ISIs and insulin secretion indexes were calculated; and their formulas are as follows:

HOMA-IR = Fins × FPG/22.5.
ISI-composite = 10,000/(mean Glu_0–120_ × mean Ins_0–120_ × FBG × Ins0)^0.5^.
HOMA-β = (20 × Ins0)/(FPG-3.5).
AUCins30/glu30 = AUCins30/AUCglu30.
AUCins120/glu120 = AUCins120/AUCglu120.
DI30 = AUCins30/glu30 × ISI-composite (Retnakaran et al., 2009).
DI120 = AUCins120/glu120 × ISI-composite (Retnakaran et al., 2009).

### Statistical analysis

Men and women have different average serum UA levels, so we analyzed these two groups separately throughout the study. Sex-specific tertiles of serum UA (μmol/L) were established in men/women as following: first tertile: ≤279.7/224.1 μmol/L, second tertile: 279.7–368.6/224.1–311.7 μmol/L, and third tertile: ≥368.8/311.7 μmol/L. All continuous variables were presented as the mean ± SD or as median and interquartile range (IQR), and all categorical variables were presented as a number (proportion). A chi-square test (χ^2^) was used for among-groups comparisons of normally distributed data for categorical variables. A Student’s *t*-test, ANOVA, or the Wilcoxon rank-sum test was used for among-groups comparisons of skewed data. Tukey’s test was performed to analyze the difference between the two groups. Lastly, the Pearson and Spearman correlation test was utilized to determine the simple correlation between serum UA levels and islet function indexes. To evaluate whether serum UA was an independent risk factor for deterioration of islet function in T2DM, we performed a stepwise multiple linear regression analysis was performed by adjusting for potential confounding factors of islet function. A two-tailed *p* ≤ 0.05 was considered as statistically significant. All statistical analyses were performed using SPSS software (version 21.0; SPSS, Chicago, IL, USA) and Origin software (version 8.0; OriginLab, Northampton, MA, USA).

## Results

The demographic and clinical characteristics of the 403 newly diagnosed T2DM patients were shown in Table 1. Briefly, there were 252 males and 151 females. The mean age of all participants was 50.21 ± 13.34 years old. The men were younger (47.97 ± 13.09 vs. 53.95 ± 12.96 years, *p* < 0.001) and had a higher prevalence of smoking (42.5% vs. 6%, *p* < 0.001) and alcohol consumption (27.8% vs. 3%, *p* < 0.001) in comparison to the women. Serum UA levels and values of BMI, DBP, FBG, BUN, and Scr were significantly higher in men than those in women (all *p* < 0.05). Compared with females, males had a significantly lower value of HDL-c (0.94 (IQR: 0.84, 1.07) vs. 1.11 (IQR: 0.95, 1.33) mmol/L, *p* < 0.001). There were no gender-specific differences in SBP, 2hPBG, TC, TG, LDL-c, HbA1c, and family history of T2DM.

**Table 1:** Clinical characteristics of participants by gender.

	Male (*n* = 252)	Female (*n* = 151)	*p*-Value
Age (years)	47.97 ± 13.09	53.95 ± 12.96	<0.01*
BMI (kg/m^2^)	25.89 ± 4.15	24.85 ± 4.21	0.016*
SBP (mmHg)	127.77 ± 16.26	131.40 ± 20.53	0.065
DBP (mmHg)	80.37 ± 11.37	77.94 ± 11.51	0.04*
FBG (mmol/L)	9.58 ± 3.5	8.85 ± 3.3	0.039*
2hPBG (mmol/L)	19.34 ± 5.49	18.50 ± 6.08	0.153
TC (mmol/L)	4.82 (4.18, 5.58)	4.87 (4.19, 5.66)	0.432
TG (mmol/L)	1.94 (1.36, 3.46)	1.84 (1.23, 2.71)	0.096
HDL-c (mmol/L)	0.94 (0.84, 1.07)	1.11 (0.95, 1.33)	<0.01*
LDL-c (mmol/L)	3.02 ± 0.83	3.02 ± 0.94	0.985
BUN (mmol/L)	5.27 ± 2.48	4.73 ± 1.40	0.013*
Scr (μmol/L)	70.05 (62.93, 18.15)	54 (46.6, 60.3)	<0.01*
UA (μmol/L)	331.05 (260.6, 400.9)	267.9 (204.7, 331.9)	<0.01*
Family history of T2DM	28 (11.3%)	20 (12.9%)	0.478
Smoking (%)	107 (42.5%)	9 (6%)	<0.01*
Consuming alcohol (%)	70 (27.8%)	5 (3.3)	<0.01*

**Notes:**

Data are expressed as mean ± SD, number (percentage), and median (interquartile ranges).

UA, uric acid; BMI, body mass index; FPG, fasting plasma glucose; PBG, postprandial plasma glucose; TC, total cholesterol; TG, triglycerides, HDL-c, high-density lipoprotein-cholesterol; LDL, low-density lipoprotein-cholesterol; BUN, blood urea nitrogen; Scr, serum creatinine; SBP, systolic blood pressure; DBP, diastolic blood pressure.

* Represented that the difference was significant.

To comprehensively evaluate islet function, insulin sensitivity, and secretion indices were calculated in all participants. As shown in Table 2, compared with females, males had significantly lower values of AUCins30/glu30, AUCins120/glu120, and DI120. The values of HOMA-IR, ISI-composite, HOMA-β, and DI30 were not significantly different between the two groups.

**Table 2:** Islet function indexes of all participants by gender.

	Male	Female	*p*-Value
HOMA-IR	3.36 (2.04, 5.32)	3.31 (1.82, 6.12)	0.801
ISI-composite	74.95 (49.24, 111.89)	65.62 (39.99, 119.63)	0.076
HOMA-β	31.52 (16.76, 71.27)	40.14 (18.37, 84.13)	0.122
AUCins30/glu30min	1.00 (0.55, 1.97)	1.40 (0.71, 2.67)	0.012*
AUCins120/glu120min	1.15 (0.63, 2.70)	2.03 (0.95, 4.64)	<0.01*
DI30	68.24 (48.82, 126.06)	81.30 (53.62, 128.23)	0.175
DI120	90.93 (52.41, 117.48)	117.06 (71.55, 243.41)	<0.01*

**Notes:**

All continuous variables were presented as median (interquartile ranges) because of skewed distribution; the difference between groups was tested by Wilcoxon rank-sum test.

* Represented that the difference was significant.

The distributions of clinical characteristics and islet function indexes of study subjects based on gender-specific tertiles of serum UA levels were presented in Tables 3 and 4, respectively. Across male serum UA tertiles, SBP, DBP, LDL-c, HDL-c, BUN, family history of T2DM, smoking, and alcohol consumption did not differ significantly while all other variables differed significantly. Specifically, compared to those in the first tertile, males in the third tertile were younger, had significantly higher BMI, increased values of TC, TG, and Scr, and more decreased values of FBG and 2hPBG. Compared with those in the second tertile, the values of TC, TG, and Scr were increased in the third tertile. Moreover, apart from age and Scr, there was no difference between the first tertile and the second tertile in terms of clinical characteristics. The analyses of islet function indexes suggested that there was no significant difference with HOMA-IR among the three tertiles; however, it did show a slightly increasing trend with increasing serum UA levels. Furthermore, HOMA-β, AUCins30/glu30, AUCins120/glu120, DI30, and DI120 all significantly increased while ISI-composite significantly decreased with ascending serum UA tertiles. In female participants, there was no significant difference with respect to age, 2hPBG, TC, LDL-c, family history of T2DM, smoking, and alcohol consumption among tertiles. The values for BMI, SBP, DBP, BUN, and Scr of the third tertile in females were significantly higher than those in the first tertile, whereas the values for FBG and HDL were significantly lower. For islet function indexes, HOMA-IR, HOMA-β, AUCins30/glu30, AUCins120/glu120, DI30, and DI120 rose significantly as serum UA increased in women. In accordance with the above results, the value of ISI-composite of the females in the third tertile was lower than that in the first tertile.

**Table 3:** Clinical characteristics and islet function indexes of male participants according to tertiles of serum uric acid levels.

	Male			
	First tertile (*n* = 84)	Second tertile (*n* = 85)	Third tertile (*n* = 83)	*p*-Value
UA (μmol/L) standard	≤279.7	279.7–368.6	≥368.8	
Age (years)	52.13 ± 11.91	46.85 ± 11.91	44.92 ± 14.40	^a^p* = 0.02, ^b^p* < 0.01, ^c^*p* = 0.6
BMI (kg/m^2^)	24.87 ± 3.27	25.94 ± 5.31	26.87 ± 3.33	^a^*p* = 0.2, ^b^p* < 0.01, ^c^*p* = 0.3
SBP (mmHg)	127.26 ± 17.2	127.33 ± 14.60	128.73 ± 17.02	^a^*p* = 1.0, ^b^*p* = 0.8, ^c^*p* = 0.8
DBP (mmHg)	79.76 ± 10.74	79.16 ± 11.25	82.22 ± 12.00	^a^*p* = 0.9, ^b^*p* = 0.3, ^c^*p* = 0.2
FBG (mmol/L)	10.35 ± 3.25	9.55 ± 3.76	8.84 ± 3.34	^a^*p* = 0.3, ^b^p* = 0.01, ^c^*p* = 0.4
2hPBG (mmol/L)	18.04 ± 5.08	16.03 ± 6.11	14.39 ± 6.29	^a^*p* = 0.1, ^b^p* < 0.01, ^c^*p* = 0.2
TC (mmol/L)	4.71 (4.06, 5.55)	4.62 (4.09, 5.48)	5.06 (4.50, 5.68)	^a^*p* = 0.7, ^b^p* = 0.04, ^c^p* = 0.01
TG (mmol/L)	1.71 (1.30, 2.35)	1.88 (1.26, 3.18)	2.65 (1.68, 4.54)	^a^*p* = 0.5, ^b^p* < 0.01, ^c^p* < 0.01
HDL-c (mmol/L)	0.94 (0.85, 1.07)	0.95 (0.84, 1.04)	0.92 (0.82, 1.09)	^a^*p* = 0.6, ^b^*p* = 0.8, ^c^*p* = 0.9
LDL-c (mmol/L)	2.99 ± 1.0	3.02 ± 0.81	2.91 ± 1.04	^a^*p* = 0.9, ^b^*p* = 0.8, ^c^*p* = 0.6
BUN (mmol/L)	4.78 ± 1.47	5.37 ± 1.67	5.65 ± 3.66	^a^*p* = 0.3, ^b^*p* = 0.06, ^c^*p* = 0.8
Scr (μmol/L)	65 (59.83, 71.08)	71.6 (65.7, 78.65)	74.9 (65.7, 87)	^a^p* < 0.01, ^b^p* < 0.01, ^c^p* < 0.01
Family history of T2DM	8 (9.5%)	9 (10.6%)	11 (13.3%)	^a^*p* = 0.8, ^b^*p* = 0.5, ^c^*p* = 0.6
Smoking (%)	36 (42.9%)	36 (42.4%)	35 (42.2)	^a^*p* = 0.9, ^b^*p* = 0.9, ^c^*p* = 1
Drinking (%)	25 (29.8)	20 (23.5%)	25 (30.1)	^a^*p* = 0.4, ^b^*p* = 0.9, ^c^*p* = 0.3
Islet function indexes				
HOMA-IR	3.14 (2.03, 4.71)	3.37 (2.08, 5.67)	3.67 (2.07, 5.82)	^a^*p* = 0.8, ^b^*p* = 0.3, ^c^*p* = 0.5
ISI-composite	85.36 (57.16, 132.7)	72.15 (47.30, 103.67)	65.56 (46.16, 95.86)	^a^*p* = 0.2, ^b^p* < 0.01, ^c^*p* = 0.1
HOMA-β	23.51 (12.70, 42.37)	34.11 (18.52, 83.75)	52.19 (21.97, 92.74)	^a^*p* = 0.1, ^b^p* < 0.01, ^c^p* = 0.02
AUCins30/glu30min	0.8 (0.46, 1.25)	1.07 (0.60, 2.25)	1.59 (0.74, 2.60)	^a^*p* = 0.2, ^b^p* < 0.01, ^c^p* < 0.01
AUCins120/glu120min	0.99 (0.43, 1.65)	1.37 (0.72, 3.28)	2.10 (0.92, 4.15)	^a^*p* = 0.1, ^b^p* < 0.01, ^c^p* < 0.01
DI30	62.87 (47.29, 95.23)	76.68 (50.21, 134.22)	87.17 (52.59, 153.97)	^a^*p* = 0.3, ^b^p* = 0.01, ^c^*p* = 0.1
DI120	69.7 (47.59, 118.58)	93.59 (53.22, 209.58)	105.30 (59.11, 220.3)	^a^*p* = 0.12, ^b^p* < 0.01, ^c^*p* = 0.1

**Notes:**

Data are expressed as mean ± SD, number (percentage) and median (interquartile ranges).

UA, uric acid; BMI, body mass index; FPG, fasting plasma glucose; PBG, postprandial plasma glucose; TC, total cholesterol; TG, triglycerides, HDL-c, high-density lipoprotein-cholesterol; LDL, low-density lipoprotein-cholesterol; BUN, blood urea nitrogen; Scr, serum creatinine; SBP, systolic blood pressure; DBP, diastolic blood pressure.

^a^*p*: first tertile vs. second tertile, ^b^*p*: first tertile vs. third teritile, ^c^*p*: second tertile vs. third tertile.

* Represented that the difference was significant.

**Table 4:** Clinical characteristics and islet function indexes of female participants according to tertiles of serum uric acid levels.

	Female			
	First tertile (*n* = 50)	Second tertile (*n* = 50)	Third tertile (*n* = 51)	*p*-Value
UA (μmol/L) standard	≤224.1	224.1–311.7	≥311.7	
Age (years)	55.1 ± 11.19	52.38 ± 13.36	54.35 ± 14.22	^a^*p* = 0.6, ^b^*p* = 0.1, ^c^*p* = 0.7
BMI (kg/m^2^)	23.53 ± 3.82	24.75 ± 4.37	26.24 ± 4.04	^a^*p* = 0.3, ^b^p* < 0.01, ^c^*p* = 0.2
SBP (mmHg)	125.62 ± 20.89	132.28 ± 18.71	136.12 ± 20.90	^a^*p* = 0.2, ^b^p* = 0.03, ^c^*p* = 0.6
DBP (mmHg)	74.68 ± 12.38	78.44 ± 10.52	80.65 ± 10.99	^a^*p* = 0.2, ^b^p* = 0.02, ^c^*p* = 0.6
FBG (mmol/L)	9.83 ± 3.63	8.57 ± 3.19	8.18 ± 1.87	^a^*p* = 0.1, ^b^p* = 0.03, ^c^*p* = 0.8
2hPBG (mmol/L)	20.02 ± 6.37	18.18 ± 5.98	17.32 ± 5.67	^a^*p* = 0.3, ^b^*p* = 0.06, ^c^*p* = 0.8
TC (mmol/L)	4.74 (4.07, 5.57)	4.83 (4.05, 5.78)	5.08 (4.40, 5.83)	^a^*p* = 0.5, ^b^*p* = 0.1, ^c^*p* = 0.4
TG (mmol/L)	1.22 (0.96, 1.79)	2.04 (1.57, 3.20)	2.17 (1.59, 3.35)	^a^p* < 0.01, ^b^p* < 0.01, ^c^*p* = 0.6
HDL-c (mmol/L)	1.24 (1.05, 1.57)	1.07 (0.95, 1.27)	1.06 (0.93, 1.21)	^a^p* < 0.01, ^b^p* < 0.01, ^c^*p* = 0.6
LDL-c (mmol/L)	3.01 ± 0.88	3.06 ± 1.07	3.0 ± 0.87	^a^*p* = 1, ^b^*p* = 1, ^c^*p* = 0.9
BUN (mmol/L)	4.45 ± 1.26	4.53 ± 1.17	5.19 ± 1.63	^a^*p* = 0.9, ^b^p* = 0.02, ^c^p* = 0.04
Scr (μmol/L)	51.1 (42.6, 56.88)	52.75 (46.58, 58.90)	58.1 (53.21, 68.6)	^a^*p* = 0.3, ^b^p* < 0.01, ^c^p* < 0.01
Family history of T2DM	8 (16%)	6 (12%)	7 (13.7%)	^a^*p* = 0.6, ^b^*p* = 0.8, ^c^*p* = 0.8
Smoking (%)	2 (4%)	4 (8%)	3 (6%)	^a^*p* = 0.4, ^b^*p* = 0.7, ^c^*p* = 0.7
Drinking (%)	3 (6%)	1 (2%)	1 (2%)	^a^*p* = 0.3, ^b^*p* = 0.3, ^c^*p* = 1
Islet function indexes				
HOMA-IR	2.56 (1.59, 5.14)	3.21 (1.25, 6.69)	3.71 (2.65, 6.81)	^a^*p* = 0.5, ^b^p* = 0.01, ^c^*p* = 0.1
ISI-composite	88.85 (48.42, 141.69)	68.50 (35.91, 143.65)	57.83 (35.72, 76.360)	^a^p* = 0.03, ^b^p* < 0.01, ^c^*p* = 0.3
HOMA-β	22.35 (9.78, 59.99)	46.15 (20.63, 86.43)	49.29 (29.17, 130.84)	^a^*p* = 0.2, ^b^p* < 0.01, ^c^*p* = 1
AUCins30/glu30min	0.81 (0.37, 1.99)	1.49 (0.72, 2.75)	1.69 (1.06, 3.18)	^a^p* = 0.03, ^b^p* < 0.01, ^c^*p* = 0.2
AUCins120/glu120min	1.23 (0.52, 3.15)	2.10 (1.06, 4.00)	2.80 (1.59, 5.62)	^a^p* = 0.04, ^b^p* < 0.01, ^c^*p* = 0.1
DI30	58.98 (46.05, 111.41)	87.62 (55.99, 132.60)	92.26 (62.56, 138.29)	^a^*p* = 0.1, ^b^p* = 0.01, ^c^*p* = 0.6
DI120	88.38 (52.34, 170.62)	112.21 (72.26, 253.54)	153.3 (99.35, 314.9)	^a^*p* = 0.1, ^b^p* < 0.01, ^c^*p* = 0.4

**Notes:**

Data are expressed as mean ± SD, number (percentage) and median (interquartile ranges).

UA, uric acid; BMI, body mass index; FPG, fasting plasma glucose; PBG, postprandial plasma glucose; TC, total cholesterol; TG, triglycerides, HDL-c, high-density lipoprotein-cholesterol; LDL, low-density lipoprotein-cholesterol; BUN, blood urea nitrogen; Scr, serum creatinine; SBP, systolic blood pressure; DBP, diastolic blood pressure.

^a^*p*: first tertile vs. second tertile, ^b^*p*: first tertile vs. third teritile, ^c^*p*: second tertile vs. third tertile.

* Represented that the difference was significant.

The correlations between serum UA levels and each islet function index by gender were exhibited in Table 5. In men, serum UA was positively associated with HOMA-β (*r* = 0.32, *p* < 0.01), AUCins30/glu30 (*r* = 0.33, *p* < 0.01), AUCins120/glu120 (*r* = 0.35, *p* < 0.01), DI30 (*r* = 0.22, *p* < 0.01), and DI120 (*r* = 0.26, *p* < 0.01), and was negatively related to ISI-composite (*r* = −0.24, *p* < 0.01). Also, serum UA was not significantly associated with HOMA-IR. In women, serum UA had positive associations with HOMA-IR (*r* = 0.22, *p* < 0.01), HOMA-β (*r* = 0.28, *p* < 0.01), AUCins30/glu30 (*r* = 0.32, *p* < 0.01), AUCins120/glu120 (*r* = 0.33, *p* < 0.01), DI30 (*r* = 0.19, *p* = 0.02), and DI120 (*r* = 0.22, *p* < 0.01) and a negative correlation with ISI-composite (*r* = −0.31, *p* < 0.01).

**Table 5:** Correlations between serum uric acid and islet function indexes by gender.

Islet function indexes	Male (*n* = 252)		Female (*n* = 151)	
	*r*	*p*-Value	*r*	*p*-Value
HOMA-IR	0.09	0.14	0.22	<0.01*
ISI-composite	−0.24	<0.01*	−0.31	<0.01*
HOMA-β	0.32	<0.01*	0.28	<0.01*
AUCins30/glu30	0.33	<0.01*	0.32	<0.01*
AUCins120/glu120	0.35	<0.01*	0.33	<0.01*
DI30	0.22	<0.01*	0.19	0.02*
DI120	0.26	<0.01*	0.22	<0.01*

**Note:**

* Represented that the *p*-value was significant.

To identify confounding factors affecting islet function, multiple linear regression analysis was performed on the above-mentioned indexes. Independent variables such as UA, age, BMI, SBP, DBP, TC, TG, HDL-c, LDL-c BUN, Scr, family history, smoking, and alcohol consumption were enrolled. In males, the regression equation of HOMA-IR was not statistically significant. The remaining regression equations exhibited significance and the results were shown in Table 6. BMI (β = −0.26, *p* < 0.01), while negatively associated with ISI-composite and Scr (β = 0.134, *p* = 0.03), solely produced a positive effect on HOMA-β. Moreover, value of AUCins30/glu30 was positively influenced by UA (β = 0.241, *p* < 0.01), BMI (β = 0.188, *p* < 0.01), DBP (β = 0.129, *p* < 0.01), and age (β = 0.128, *p* < 0.01). Similarly, AUCins120/glu120 was jointly affected by the four factors mentioned above (β = 0.334, 0.169, 0.174, 0.122, respectively) and was negatively affected by TG (β = −0.151, *p* < 0.01). Additionally, UA, SBP, and Scr were positive influencing factors for both DI30 (β = 0.216, 0.123, 0.158, respectively) and DI120 (β = 0.272, 0.139, 0.149, respectively), while serum TC was a negative influencing factor for both of the indexes (β = −0.185 and β = −0.181, respectively). As shown in Table 7, no variable entered into the equation of HOMA-β and DI30 in women. Notably, serum UA concentration independently affected AUCins30/glu30 (β = 0.263, *p* < 0.01), AUCins120/glu120 (β = 0.296, *p* < 0.01), and DI120 (β = 0.191, *p* = 0.03), while BMI independently affected HOMA-IR (β = 0.308, *p* < 0.01) and ISI-composite (β = −0.422, *p* < 0.01).

**Table 6:** Multiple linear regression analysis on related variables for islet function indexes in men.

Islet function indexes	Partial regression coefficient (B)	Standard error (SE)	Standard partial regression coefficient (β)	*p*-Value
HOMA-IR	No variable were entered into the equation			
ISI-composite				
BMI	−4.36	1.02	−0.26	<0.01
HOMA-β				
Scr	1.48	0.69	0.134	0.03
AUCins30/glu30				
UA	0.004	0.001	0.241	<0.01
BMI	0.077	0.025	0.188	<0.01
DBP	0.019	0.009	0.129	0.03
Age	0.017	0.008	0.128	0.04
AUCins120/glu120				
UA	0.007	0.001	0.334	<0.01
DBP	0.037	0.012	0.174	<0.01
TG	−0.117	0.045	−0.151	0.01
BMI	0.098	0.035	0.169	<0.01
Age	0.023	0.011	0.122	0.04
DI30				
UA	0.163	0.048	0.216	<0.01
TC	−11.84	3.8	−0.185	<0.01
Scr	0.841	0.338	0.158	<0.01
SBP	0.63	0.303	0.123	0.04
DI120				
UA	0.346	0.08	0.272	<0.01
TC	−19.58	6.31	−0.181	<0.01
SBP	1.205	0.514	0.139	0.02
Scr	1.327	0.562	0.149	0.02

**Notes:**

Categorical variables were family history (dichotomous: yes = 1, no = 0), smoking (dichotomous: yes = 1, no = 0), and drinking (dichotomous: yes = 1, no = 0). *p*-Value derived from stepwise multiple linear regression analysis. Only the variables whose *p* < 0.05 were shown in the table.

UA, uric acid; BMI, body mass index; TC, total cholesterol; TG, triglycerides; Scr, serum creatinine; SBP, systolic blood pressure; DBP, diastolic blood pressure.

**Table 7:** Multiple linear regression analysis on related variables for islet function indexes in women.

Islet function indexes	Partial regression coefficient (B)	Standard error (SE)	Standard partial regression coefficient (β)	*p*-Value
HOMA-IR				
BMI	0.215	0.054	0.308	<0.01
ISI-composite				
BMI	−6.22	1.09	−0.422	<0.01
HOMA-β	No variable were entered into the equation			
AUCins30/glu30				
UA	0.004	0.001	0.263	<0.01
AUCins120/glu120				
UA	0.009	0.002	0.296	<0.01
DI30	No variable were entered into the equation			
DI120				
UA	2.844	1.302	0.191	0.03

**Notes:**

Categorical variables were family history (dichotomous: yes = 1, no = 0), smoking (dichotomous: yes = 1, no = 0) and drinking (dichotomous: yes = 1, no = 0). *p*-Value derived from stepwise multiple linear regression analysis. Only the variables whose *p* < 0.05 were shown in the table.

UA, uric acid; BMI, body mass index.

Multivariable linear regression analysis indicated that BMI also played an important role in islet function both in men and women. All participants were divided into three groups: normal weight, overweight, and obesity, followed by calculation of the seven islet function indexes. As shown in Table 8, the values of serum UA levels, HOMA-IR, HOMA-β, AUCins30/glu30, AUCins120/glu120, and DI30 of the obesity group in males were significantly higher than those in the normal weight group, whereas the value ISI-composite was significantly lower. In female participants, serum UA levels, HOMA-IR, HOMA-β, AUCins30/glu30, AUCins120/glu120, DI30, and DI120 all significantly increased, while ISI-composite significantly decreased with increasing BMI values.

**Table 8:** Serum uric acid and islet function indexes of all participants according to BMI in male and female.

	Male				Female			
	Normal weight (BMI < 23 kg/m^2^)	Overweight (BMI: 23–24.9 kg/m^2^)	Obesity BMI ≥ 25 kg/m^2^	*p*-Value	Normal weight (BMI < 23 kg/m^2^)	Overweight (BMI: 23–24.9 kg/m^2^)	Obesity (BMI ≥ 25 kg/m^2^)	*p*-Value
UA (μmol/L)	300.2 (226.6, 251.7)	279.7 (227.4, 343.4)	359.1 (280.6, 438.9)	^a^*p* = 0.3; ^b^*p* = 0.001*; ^c^*p* < 0.001*	250.4 (193.5, 303.7)	249.8 (203.1, 318.8)	299.8 (227.9, 404.7)	^a^*p* = 0.4; ^b^*p* < 0.001*; ^c^*p* = 0.02*
Islet function indexes								
HOMA-IR	2.1 (1.5, 4.4)	3.1 (2.0, 4.1)	4.0 (2.4, 6.3)	^a^*p* = 0.06; ^b^*p* < 0.001*; ^c^*p* = 0.008*	2.7 (1.3, 4.0)	2.9 (1.6, 4.8)	5.1 (2.7, 7.4)	^a^*p* = 0.7; ^b^*p* < 0.001*; ^c^*p* = 0.005*
ISI-composite	103.4 (71.2, 154.3)	86.1 (65.2, 116.7)	61.9 (45.3, 98.3)	^a^*p* = 0.3; ^b^*p* < 0.001*; ^c^*p* = 0.001*	89.5 (61.7, 157.7)	68.8 (52.4, 118.6)	44.1 (32.6, 75.4)	^a^*p* = 0.1; ^b^*p* < 0.001*; ^c^*p* < 0.001*
HOMA-β	19.2 (10.8, 36.2)	26.1 (13.5, 44.1)	42.7 (20.2, 88.6)	^a^*p* = 0.1; ^b^*p* < 0.001*; ^c^*p* = 0.001*	27.1 (11.7, 43.6)	39.7 (14.7, 75.3)	66.3 (28.1, 138.3)	^a^*p* = 0.1; ^b^*p* < 0.001*; ^c^*p* = 0.007*
AUCins30/glu30min	0.6 (0.4, 1.1)	0.8 (0.5, 1.2)	1.3 (0.8, 2.3)	^a^*p* = 0.2; ^b^*p* < 0.001*; ^c^*p* < 0.001*	0.8 (0.4, 1.7)	1.2 (0.5, 2.0)	2.4 (1.2, 3.3)	^a^*p* = 0.2; ^b^*p* < 0.001*; ^c^*p* = 0.002*
AUCins120/glu120min	0.8 (0.4, 1.6)	1.0 (0.5, 1.6)	1.7 (0.8, 3.5)	^a^*p* = 0.5; ^b^*p* < 0.001*; ^c^*p* = 0.001*	1.2 (0.5, 2.1)	2.1 (0.9, 4.4)	3.6 (1.5, 5.6)	^a^*p* = 0.03*; ^b^*p* < 0.001*; ^c^*p* = 0.01*
DI30	59.7 (43.2, 114.4)	62.9 (43.2, 95.9)	82.1 (52.1, 146.6)	^a^*p* = 0.9; ^b^*p* = 0.03*; ^c^*p* = 0.01*	71.0 (46.7, 100.3)	81.3 (53.7, 134.7)	93.5 (56.2, 158.6)	^a^*p* = 0.3; ^b^*p* = 0.03*; ^c^*p* = 0.4
DI120	70.3 (48.3, 206.5)	76.8 (48.2, 124.7)	101.4 (55.4, 215.2)	^a^*p* = 0.8; ^b^*p* = 0.2; ^c^*p* = 0.04*	91.2 (53.7, 159.6)	135.9 (65.2, 313.0)	153.7 (83.8, 255.9)	^a^*p* = 0.1; ^b^*p* = 0.008*; ^c^*p* = 0.5

**Notes:**

Data are expressed as median (interquartile ranges).

^a^*p*: first tertile vs. second tertile, ^b^*p*: first tertile vs. third teritile, ^c^*p*: second tertile vs. third tertile.

* Represented that the difference was significant.

## Discussion

In this population-based study of newly diagnosed T2DM in Chinese participants, we found that the values of AUCins30/glu30 and AUCins120/glu120 were higher in females than in males. AUCins/glu denotes the levels of insulin secretion and DI represents the ability of islet β-cells to compensate against insulin resistance. There was no significant difference between men and women in insulin sensitivity. In agreement with our results, a previous study demonstrated higher insulin secretion in women and similar insulin sensitivity among women and men of a normal glucose tolerance population (Clausen et al., 1996). This phenomenon may be explained by the ability of female hormones to facilitate the adaptive increase of insulin biosynthesis, glucose stimulated insulin secretion and islet β–cell mass (Nadal et al., 2009).

Theoretically, increased insulin secretion could compensate for insulin resistance and could maintain normal plasma glucose levels for 10 years before the onset of clinical T2DM (Reaven, 2009). There are two phases of insulin secretion that occur in individuals with normal glucose tolerance: the early phase and the basal phase. Early-phase insulin secretion declines and even disappears in T2DM patients, suggesting that early-phase insulin secretion is very sensitive to glucotoxicity (Kahn et al., 2001). Moreover, the degree of basal insulin secretion impairment is consistent with increased plasma glucose levels, indicating that basal insulin secretion is the main influencing factor on the plasma glucose levels (Lin et al., 2010). Our results that the value of DI120 in females was higher than that in males suggested a slower deterioration of β-cells in females. This might explain why the control of FBG levels in women is superior to that in men.

Hyperuricemia has been regarded as an independent risk factor for cerebrovascular disease, and it is also a powerful and optimal predictor for cardiovascular events in diabetic patients (Verdoia et al., 2014). Still, an independent relationship between serum UA and islet dysfunction has never been confirmed. In our study, insulin resistance and insulin secretion increased with rising serum UA levels in both men and women. After adjusting for confounding factors influencing islet function, serum UA was associated with AUCins30/glu30, AUCins120/glu120, DI30, and DI120 in males. Meanwhile, serum UA independently affected AUCins30/glu30, AUCins120/glu120, and DI120 in females. Together, these results indicate that serum UA plays a more important role in augmenting insulin secretion rather than in insulin resistance.

With regards to the mechanism underlying the effect of serum UA on insulin resistance and insulin secretion, there is no definite explanation despite numerous animal and clinical studies. Previous studies have suggested that hyperuricemia and insulin resistance mutually promote each other. Specifically, hyperinsulinemia facilitates the reabsorption of urate and Na^+^ in the brush border membranes of the renal proximal tubule (Enomoto et al., 2002). In addition, high serum insulin could reduce the clearance rate of urate and caused imbalances in human purine metabolism by increasing hepatic de-novo lipogenesis (Flannery et al., 2012). In the present study, the levels of Scr were increased with rising serum UA tertiles, showing that insulin resistance decreased urate renal clearance. In turn, it is acceptable that oxidative stress and inflammation can also be a source of hyperuricemia, which subsequently worsens insulin resistance. Hyperuricemia leads to the reduction of endogenous nitric oxide and the injury of endothelial cells, which directly results in oxidative stress and inflammation of adipocytes and is involved in insulin resistance (Feron et al., 1999). It has been reported that hyperuricemia inhibits proliferation of islet β-cells and induces oxidative damage by activating adenosine monophosphate-activated protein kinase and extracellular signal-regulated kinase signal pathways (Zhang et al., 2013). In consideration of the association between serum UA and insulin secretion, some studies have reported that high serum UA impairs insulin secretion in rat models (Scott et al., 1981; Rocic et al., 2005), however, more researches including our study conclude that serum UA augments insulin secretion, particularly basal insulin secretion. These results may be interpreted as existed differences between rodents and humans. Hyperuricemia induces increased insulin secretion to compensate for insulin resistance. There is a study finding that hyperuricemic patients have increased levels of atrial natriuretic peptide (ANP) and brain natriuretic peptide, hence, the beneficial effect of ANP levels on islet β-cell could not be excluded (Hermans, Ahn & Rousseau, 2009). Furthermore, antioxidant properties of serum UA protect islet β-cell function from oxidative stress, which might, thereby, improve islet neogenesis and islet cell apoptosis. As many studies have been undertaken to obtain a better understanding of the complex molecular mechanisms inducing insulin secretion and insulin resistance (Ohneda, Ee & German, 2000; Arcidiacono et al., 2014), the effect of serum UA on potential target genes and pathways of insulin production will provide novel research directions for our further investigation.

In this study, we also observed that BMI showed significant differences among the three UA tertiles in both men and women. As shown in Table 8, with the obese cohort, insulin resistance was more evident than that in the normal weight group, in spite of the more evident two-phase insulin secretion. These results are compatible with the hyperinsulinemic conditions that characterize insulin resistance states. However, consistent with our results, another study has showed a greater potential in the recovery of islet function in part of obese population (Shetty et al., 2012). Consequently, for obese T2DM patients, the primary goal is to improve insulin sensitivity by weight loss. Previous studies have reported that improvement of insulin sensitivity and delay of progression of T2DM could be observed after a modest calorie restriction and weight reduction (Greco et al., 2014; Grams & Garvey, 2015). In addition, in males, TC and TG negatively affected insulin secretion indexes. An eight-year longitudinal study involving nonelderly people has showed that dyslipidemia and high blood pressure are associated with insulin resistance, and VLDL-cholesterol are correlated with impaired early phase insulin secretion (Kekalainen, Sarlund & Laakso, 2000).

There were several limitations in this study. First, there were limitations to the analysis of disease risk from a causal relationship. Second, potential variables, which would affect islet function, such as exercise, education, profession, and energy intake, were not taken into consideration. However, the large-scale population-based ensured convincing outcomes of investigating the interaction of the serum UA and islet function, and we speculated about potential molecular mechanisms by which UA affects islet function. Second, to the best of our knowledge, it was the first study that used seven indexes to comprehensively reflect the different phases of insulin secretion and insulin sensitivity. Third, we also analyzed the other risk factors influencing islet function.

## Conclusion

Serum UA harbored a positive correlation with insulin secretion and insulin resistance indexes in newly diagnosed T2DM patients, which was influenced by age, BMI, and serum lipids. Hence, serum UA may be considered as a predictor for islet function in clinical settings.

## Supplemental Information

10.7717/peerj.4515/supp-1 Supplemental Information 1 Clinical characteristics and the results of OGTT and insulin release test of all participants.

## Additional Information and Declarations

### Data Availability

The following information was supplied regarding data availability: The raw data are provided in a Supplemental Dataset Files.
